# Central Nervous System Vasculitis Secondary to Sarcoidosis: A Rare Case of Lupus Pernio With Complete Occlusion of Right Internal Carotid Artery

**DOI:** 10.7759/cureus.10274

**Published:** 2020-09-06

**Authors:** Saeed Arif, Shaheer Arif, Jahanzeb Liaqat, Atiq-ur-Rehman Slehria, Abdur Rahim Palwa

**Affiliations:** 1 Neurology, Pak-Emirates Military Hospital, Rawalpindi, PAK; 2 Radiology, Armed Forces Institute of Radiology and Imaging, Rawalpindi, PAK

**Keywords:** central nervous system vasculitis, digital subtraction angiography, internal carotid artery occlusion, lupus pernio, neurosarcoidosis, sarcoidosis, non-caseating granuloma

## Abstract

Sarcoidosis is a systemic inflammatory disorder resulting from an inappropriate immune response to ubiquitous environmental stimuli. It has a predilection for African Americans and people of Northern European countries. The classic histology is that of a non-caseating granuloma. Central nervous system involvement is a rare occurrence in sarcoidosis and even in this manifestation, the presence of vasculitis is comparatively uncommon.

We present a case of a 35-year-old female, who presented with complaints of persistent headache of moderate intensity and had a violaceous plaque on nose, being treated by a dermatologist. The patient on further workup had mildly raised proteins on cerebrospinal fluid analysis. MRI brain showed multiple foci in bilateral frontoparietal regions and centrum semiovale, while digital subtraction angiography brain depicted vasculitis of small vessels of brain and complete occlusion of right internal carotid artery at its origin. Biopsy of lesion on nose was performed that showed chronic granulomatous inflammation. A diagnosis of brain vasculitis secondary to sarcoidosis was made. The patient was treated with plasmapheresis and pulse steroid therapy initially, and later on with cyclophosphamide and azathioprine. This resulted in resolution of headache and nose lesion.

## Introduction

Sarcoidosis is an idiopathic multisystem inflammatory disorder that occurs probably due to an exaggerated immune reaction to ubiquitous environmental stimuli [1]. It most commonly affects the lymph nodes, lungs, and skin [2]. The incidence of sarcoidosis is greatest in African Americans and Northern European countries [1]. The classical histological picture is that of a non-caseating granuloma of epithelioid histiocytes and giant cells surrounded by lymphocytes but can have a necrotizing pattern as well [3]. The clinical presentation includes general symptoms, such as weight loss, fatigue, and night sweats along with system-specific symptoms involved by sarcoidosis [1].

The nervous system is involved in about 5% of cases of sarcoidosis [4]. In cases of neurosarcoidosis (NS), less than one-fourth of patients may have non-neurological symptoms [5]. NS can involve various structures including the cranial nerves, hypothalamus, pituitary, brain parenchyma, meninges, and spinal cord. Cerebrovascular manifestations tend to be rare with large vessel vasculitis being the least common [6]. We present a case of central nervous system (CNS) vasculitis as an expression of NS in a patient with lupus pernio.

## Case presentation

A 35-year-old female patient reported to the Department of Neurology, Pak-Emirates Military Hospital with headache for the past three months. Headache was progressive, holocranial, and moderate in intensity and was relieved only after taking analgesics. Headache increased in intensity with time. However, headache-associated symptoms like nausea, vomiting, motion sensitivity, and relationship to body posture were not present. She denied any head trauma, seizures, and blurring of vision during or before the start of headache and never lost consciousness. The systemic review was unremarkable for fever, weight loss, night sweats, joint pains, rash, genital ulcers, and xerostomia. 

Previously, she was under dermatologist treatment with topical preparations for a single non-resolving violaceous plaque on the nose for a couple of weeks and was referred to the Department of Neurology for evaluation of persistent headache. The patient denied having any chronic neurological and rheumatological illness. Moreover, the patient never had raised blood pressure. There was no history of any vasculitic or rheumatological disease in first-degree relatives. The patient was not an addict and a non-smoker.

On examination, the patient was vitally stable with blood pressure 125/85 mmHg, pulse 74 beats/minute, respiratory rate 19 breaths/minute, and she was afebrile. Orthostatic hypotension was absent with the patient’s blood pressure being maintained on standing up from supine position. Clinical examination of face was normal except for a violaceous plaque on nose. Parotid glands were not enlarged. Moreover, fundoscopy of retina did not reveal any pathology. Systemic examination including chest and abdomen was also unremarkable. Sensory and motor systems were intact bilaterally in both upper limbs and lower limbs. All the cranial nerve functions were intact.

**Figure 1:**
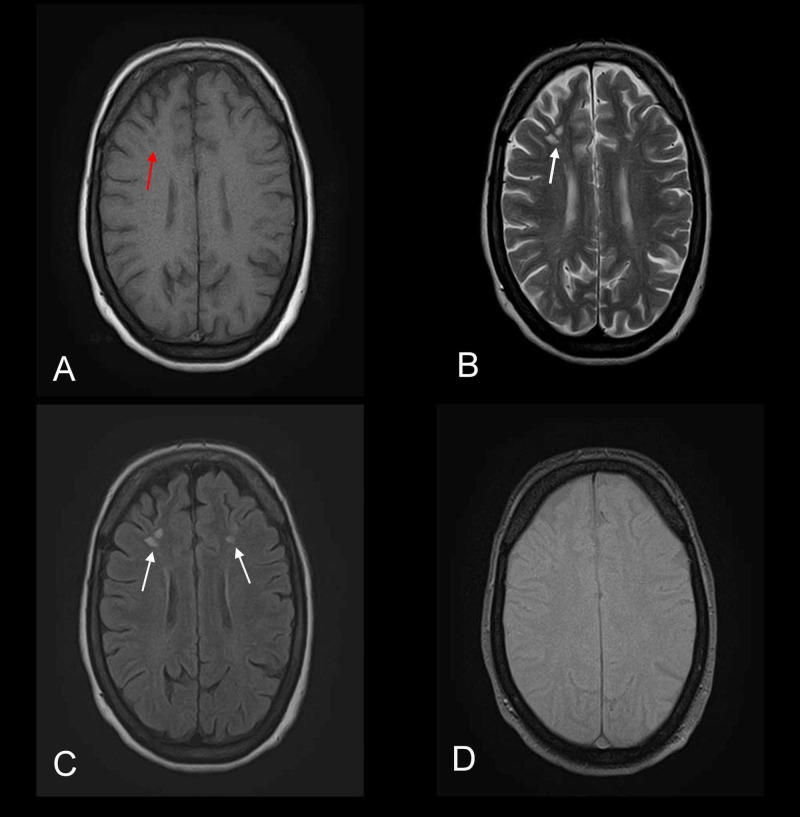
Axial MR images depicting microangiopathic ischemic changes consistent with small vessel pathology Multiple foci are noted distributed bilaterally in the frontal regions. (A) Foci appear hypointense on axial T1-weighted MR image (red arrow). (B and C) Same foci appear hyperintense on axial T2-weighted and FLAIR MR images (white arrows). (D) GRE sequence does not show any bleed. MR, magnetic resonance; FLAIR, fluid-attenuated inversion recovery; GRE, gradient-echo.

**Figure 2:**
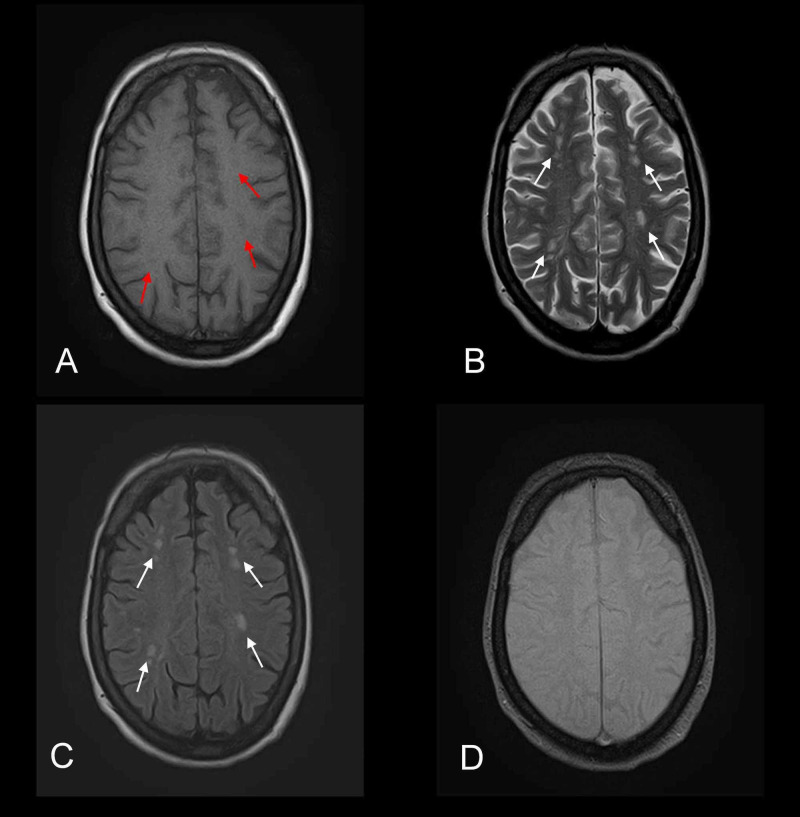
Axial MR images depicting microangiopathic ischemic changes consistent with small vessel pathology Multiple foci are noted distributed bilaterally in the frontoparietal regions and centrum semiovale. (A) Foci appear hypointense on axial T1-weighted MR image (red arrows). (B and C) Same foci appear hyperintense on axial T2-weighted and FLAIR MR images (white arrows). (D) GRE sequence does not show any bleed. MR, magnetic resonance; FLAIR, fluid-attenuated inversion recovery; GRE, gradient-echo.

**Figure 3:**
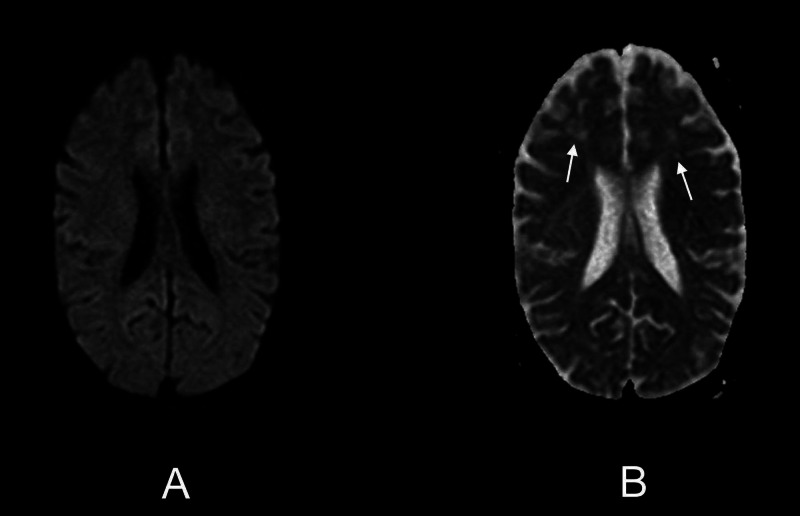
Axial MR images showing DW and ADC sequences Multiple foci are noted distributed bilaterally in the frontoparietal regions. No diffusion restriction is demonstrated on (A) axial DW MR image and (B) axial ADC MR image (white arrows). MR, magnetic resonance; DW, diffusion-weighted; ADC, apparent diffusion coefficient.

Urgent non-contrast CT brain was unremarkable. MRI brain demonstrated multiple lesions in bilateral frontoparietal regions and centrum semiovale (Figures 1, 2). Lesions did not show restricted diffusion on diffusion-weighted and apparent diffusion coefficient MR sequences (Figure 3). These lesions were considered as microangiopathic ischemic changes consistent with small vessel disease or pathology. Cerebrospinal fluid (CSF) analysis showed a normal opening pressure of 180 mmHg and revealed raised proteins 65 mg/dl without any cells. Mycobacterium tuberculosis polymerase chain reaction (PCR), Venereal Disease Research Laboratory (VDRL) test, and cryptococcal antigen were negative in CSF. Human herpes simplex virus I and II PCR, Cytomegalovirus PCR, and Epstein-Barr virus PCR were also negative in CSF.

**Figure 4:**
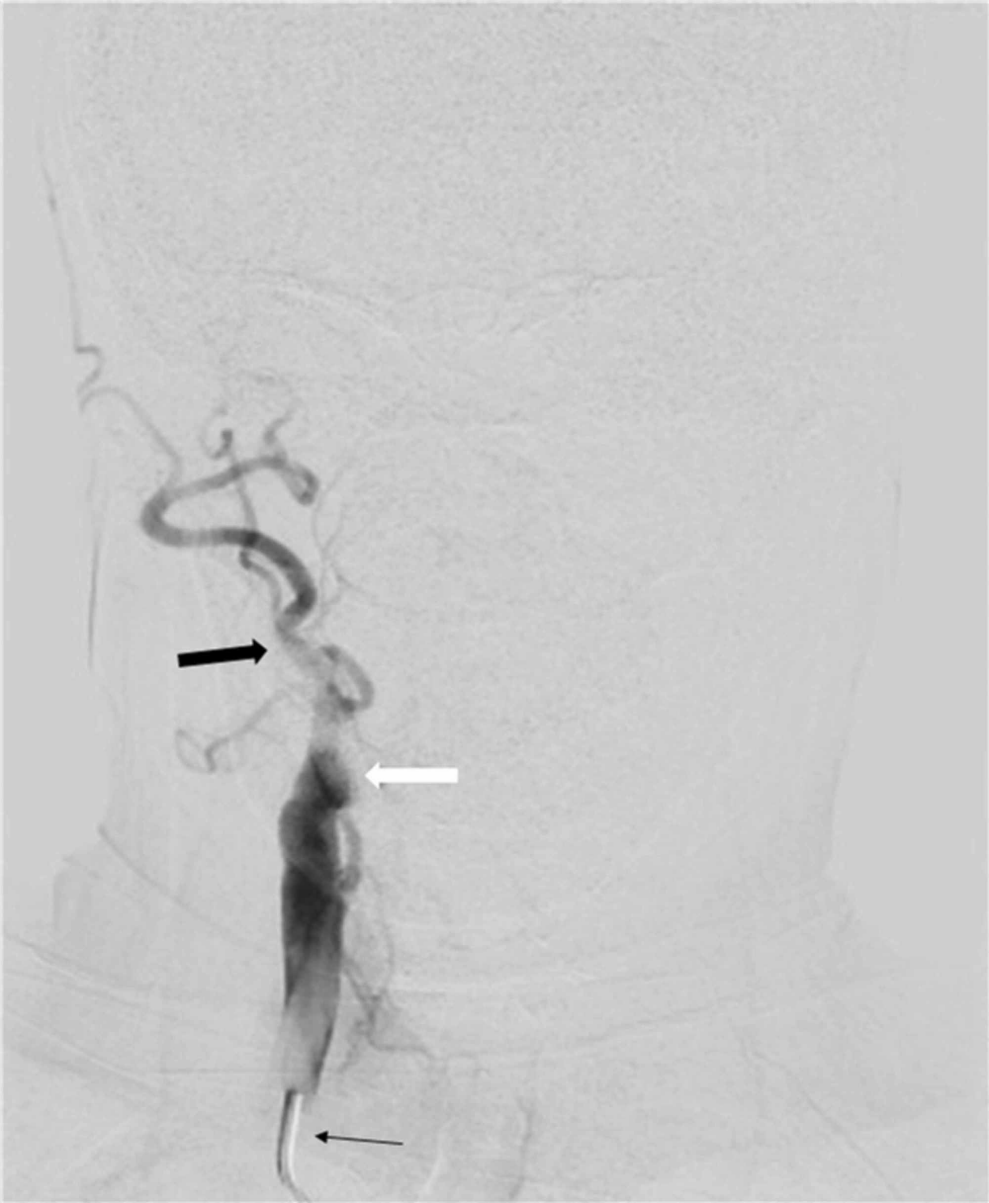
Right carotid artery angiogram AP view on DSA brain Position of catheter is visible in right common carotid artery (thin black arrow). Complete cut-off of contrast flow is noted at origin of right internal carotid artery (thick white arrow); however, contrast flow is diverted to right external carotid artery (thick black arrow). DSA, digital subtraction arteriography; AP, anteroposterior.

**Figure 5:**
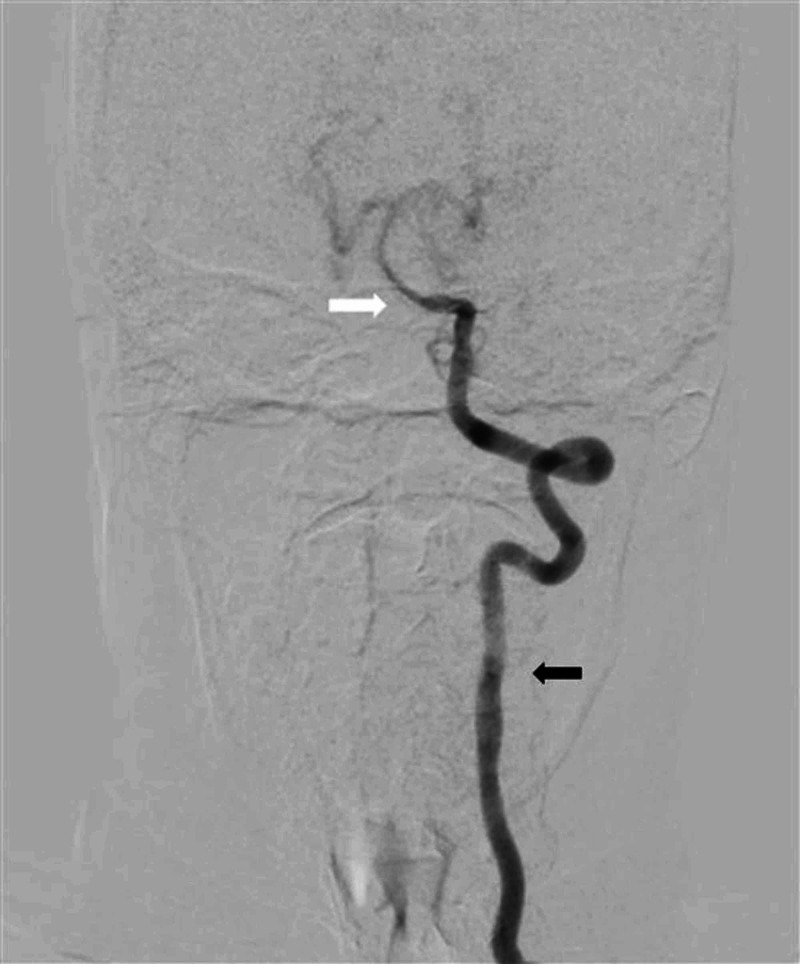
Left vertebral artery angiogram AP view on DSA brain Normal contrast flow in the left vertebral artery is visible (thick black arrow); however, basilar artery through out its course is attenuated with reduced contrast flow (thick white arrow). DSA, digital subtraction arteriography; AP, anteroposterior.

**Figure 6:**
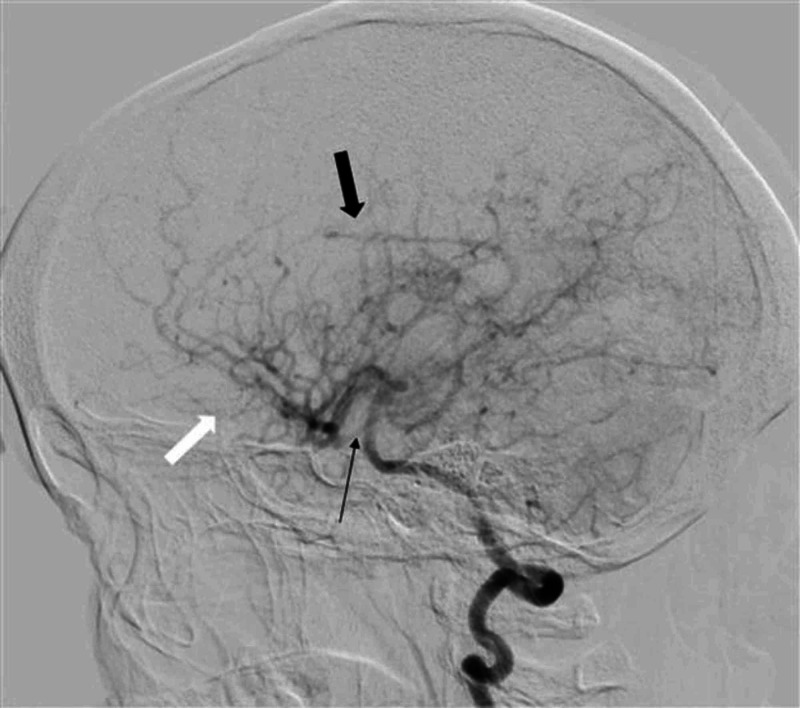
Left vertebral artery angiogram lateral view on DSA brain Right middle cerebral artery with its branches (thick black arrow) and right anterior cerebral artery with its branches (thick white arrow) are being filled with retrograde contrast flow from right posterior communicating artery (thin black arrow). DSA, digital subtraction arteriography.

**Figure 7:**
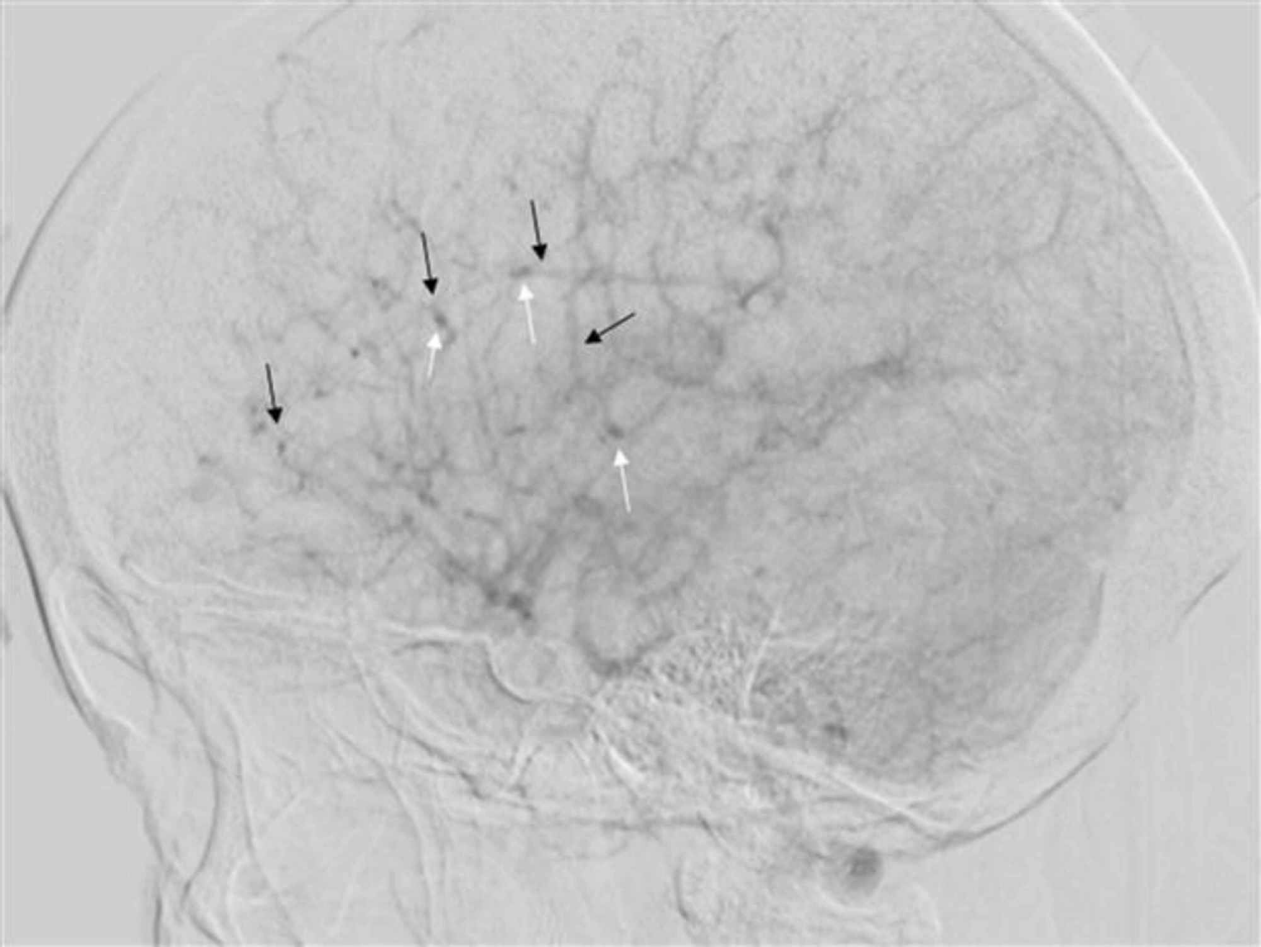
Lateral view showing branches of MCA and ACA on DSA brain MCA and ACA are being supplied from posterior communicating artery on left vertebral artery angiogram. Multifocal areas of narrowing (thin black arrows) and dilatation (thin white arrows) are seen in the distal branches of MCA and ACA. This being consistent with findings of vasculitis. DSA, digital subtraction angiography; MCA, middle cerebral artery; ACA, anterior cerebral artery.

Keeping in view the young age of patient, raised CSF proteins, and microangiopathic ischemic changes on MRI brain in the absence of systemic hypertension, conventional digital subtraction angiography (DSA) brain was performed without considering the need for magnetic resonance arteriography and venography. On performing DSA brain, the angiogram of right carotid artery showed complete cut-off of contrast flow in right internal carotid artery at its origin, with total contrast flow diverted into right external carotid artery (Figure 4). An area of focal attenuation in the basilar artery was also noted on left vertebral artery angiogram (Figure 5). Moreover, left vertebral angiogram also demonstrated that right middle cerebral artery (MCA) and right anterior cerebral artery (ACA) were being filled with retrograde contrast flow from right posterior communicating artery (Figure 6). Distal branches of bilateral MCA and ACA revealed multifocal areas of stenosis and dilatation (Figure 7). Right vertebral artery was hypoplastic representing a normal anatomical variant. The findings of DSA brain were considered to be consistent with the vasculitis of brain vessels.

The complete vasculitic screen was unremarkable, including antinuclear antibodies, anti-double-stranded-DNA antibodies, anti-phospholipid antibodies, both C and P anti-neutrophilic cytoplasmic antibodies, extractable nuclear antigen antibodies, anti-tissue transglutaminase antibodies, anti-endomysial antibodies, and anti-thyroid antibodies. Angiotensin-converting enzyme (ACE) levels were raised measuring 225 U/l (normal up to 45 U/l). Complement levels were within normal limits. Paraneoplastic autoimmune screen was also unremarkable. Contrast-enhanced CT chest, abdomen, and pelvis was unremarkable. Spirometry did not reveal any restrictive or obstructive lung dysfunction. Transthoracic echocardiography was also normal.

At this stage, the biopsy of nose lesion was carried out, and biopsy specimen revealed non-caseating granulomatous inflammation likely consistent with sarcoidosis. At this point, a multidisciplinary approach was used, involving radiologist, neurologist, rheumatologist, histopathologist, and neurosurgeon, to reach the final diagnosis of CNS vasculitis secondary to sarcoidosis, and it was decided not to perform an invasive brain biopsy.

The patient underwent five sessions of plasmapharesis with five doses of one-gram intravenous methylprednisolone. Considering severe nature of brain vasculitis, immediate immunosuppression with cyclophosphamide was started along with intermittent regime of steroids. In total eight grams of cyclophosphamide over six months was injected. The patient's headache settled and nose lesion resolved with treatment. Long-term immunosuppression after cyclophosphamide was maintained with azathioprine 3 mg/kg body weight. Over the period of next six months, the patient became symptom-free.

## Discussion

Neurological involvement in sarcoidosis is uncommon. It may present as a diagnosed case of sarcoidosis presenting with neurological symptoms or can be there when first diagnosis of sarcoidosis is made [7,8]. NS clinically presents as cranial nerve deficits, headache, ataxia, cognitive malfunction, weakness, and seizures in descending order of frequency [9]. Our patient had ongoing lupus pernio that was missed owing to the presence of only a single lesion over the nose. She presented with headache as the sole manifestation of neurological involvement, and further workup lead to the diagnosis of probable NS according to the criteria of Zajicek et al. [10].

Vascular involvement in sarcoidosis is unusual with African American and Asian patients making up a large proportion of those with large vessel vasculitis [11]. Etiologies of CNS vasculitis including infectious, autoimmune, and systemic inflammatory diseases must be ruled out by history, clinical examination, and appropriate investigations when suspecting sarcoidosis as the cause [12].

Small vessel vasculitis of perforating brain arteries is the usual picture in NS [6]. Our patient had small vessel vasculitis involving the branches of MCA and ACA depicted by dilatations and stenoses on DSA. The proportion of ischemic insults in brain as compared to intracranial vasculitis found on postmortem examination is small [6]. This being a paradoxical finding. Infarcts in NS are usually small in size and occur in deep brain substance though large infarcts can also occur [13]. The incidence of hemorrhage is reported to be 0.6% in NS case series with the majority occurring supratentorially [14].

Distal part of internal carotid artery involvement in NS resembling moyamoya vasculopathy has been previously reported [15-17]. Stenosis of left internal carotid in NS was described by Macêdo et al. [13]. However, in our case proximal right internal carotid artery was completely occluded at its origin with complete diversion of contrast flow to external carotid artery. This to our knowledge is the first report of proximal internal carotid occlusion in a case of sarcoidosis.

A definitive diagnosis of NS is made on brain biopsy. This is not possible in all cases, and therefore usually a probable diagnosis is made based upon clinical syndrome suggesting NS with evidence of CNS inflammation by CSF analysis and/or MRI and exclusion of alternate diagnoses with evidence of systemic sarcoidosis [10]. Diagnosis of CNS vasculitis itself is made difficult with low specificity and sensitivity of CSF analysis and MRI brain findings. This dilemma is further amplified if clinical or radiological presentation is atypical [18]. Even DSA has only around 60% sensitivity in diagnosing vasculitis [19]. Moreover, the availability of such procedures at only a handful of centers in developing countries with limited health resources makes the situation cumbersome for treating neurologist. Therefore, threshold for treating CNS vasculitis secondary to sarcoidosis can be low, particularly when a patient has systemic manifestations and then presents with neurological features, suggesting CNS involvement, till the time new diagnostic tests with better sensitivity and specificity are developed for definitive diagnosis of CNS vasculitis.

Treatment of NS is rooted mainly in corticosteroids with alternate immunosuppressive therapies in resistant and severe cases [1]. Infliximab can be instituted in patients refractory to glucocorticoid therapy [20]. The prognosis of patients depends upon the extent of CNS involvement. In our case, parenchymal involvement was minimal with the absence of hydrocephalus. Vascular obstruction had been circumvented with adequate collateral development. Aggressive treatment was initiated before serious manifestations arose, which resulted in the patient responding to treatment and resolution of symptoms.

## Conclusions

Cerebrovascular involvement in NS though rare as compared to its other manifestations carries high morbidity and mortality. Vasculitis if present usually involves small vessels resulting in ischemic strokes though hemorrhage may also occur. There can be involvement of large arteries as well, and complete occlusion of internal carotid artery at its origin was present in our case. In working up cases of CNS vasculitis, it is prudent to rule out all other causes before diagnosing it as NS. Prompt and aggressive treatment institution can prevent grave complications from developing and lead to better clinical outcomes.

## References

[1] Iannuzzi MC, Rybicki BA, Teirstein AS (2007). Sarcoidosis. N Engl J Med.

[2] Bathla G, Singh AK, Policeni B, Agarwal A, Case B (2016). Imaging of neurosarcoidosis: common, uncommon, and rare. Clin Radiol.

[3] Rosen Y (2015). Four decades of necrotizing sarcoid granulomatosis: what do we know now?. Arch Pathol Lab Med.

[4] Burns TM (2003). Neurosarcoidosis. Arch Neurol.

[5] Joseph FG, Scolding NJ (2009). Neurosarcoidosis: a study of 30 new cases. J Neurol Neurosurg Psychiatry.

[6] Bathla G, Watal P, Gupta S, Nagpal P, Mohan S, Moritani T (2018). Cerebrovascular manifestations of neurosarcoidosis: an underrecognized aspect of the imaging spectrum. AJNR Am J Neuroradiol.

[7] Eid H, O'Connor CR, Catalano E, Reginato AJ (1998). Life-threatening vasculitis associated with sarcoidosis. J Clin Rheumatol.

[8] Michotte A, Dequenne P, Jacobovitz D, Hildebrand J (1991). Focal neurological deficit with sudden onset as the first manifestation of sarcoidosis: a case report with MRI follow-up. Eur Neurol.

[9] Scott TF, Yandora K, Valeri A, Chieffe C, Schramke C (2007). Aggressive therapy for neurosarcoidosis: long-term follow-up of 48 treated patients. Arch Neurol.

[10] Zajicek JP, Scolding NJ, Foster O (1999). Central nervous system sarcoidosis—diagnosis and management. QJM.

[11] Fernandes SRM, Singsen BH, Hoffman GS (2000). Sarcoidosis and systemic vasculitis. Semin Arthritis Rheum.

[12] Kraemer M, Berlit P (2010). Systemic, secondary and infectious causes for cerebral vasculitis: clinical experience with 16 new European cases. Rheumatol Int.

[13] Macêdo PJOM, da Silveira VC, Ramos LT, Nóbrega FR, Vasconcellos LFR (2016). Isolated central nervous system vasculitis as a manifestation of neurosarcoidosis. J Stroke Cerebrovasc Dis.

[14] O'Dwyer JP, Al-Moyeed BA, Farrell MA (2013). Neurosarcoidosis-related intracranial haemorrhage: three new cases and a systematic review of the literature. Eur J Neurol.

[15] Kim JS, No YJ (2006). Moyamoya-like vascular abnormality in pulmonary sarcoidosis. Cerebrovasc Dis.

[16] Ko JK, Lee SW, Choi CH (2009). Moyamoya-like vasculopathy in neurosarcoidosis. J Korean Neurosurg Soc.

[17] Takenaka K, Ito M, Kumagai M (1998). Moyamoya disease associated with pulmonary sarcoidosis: case report. Neurol Med Chir (Tokyo).

[18] Arif S, Liaqat J, Nawaz KH, Hashmat A (2017). Focal hemispheric central nervous system vasculitis: an unusual form of primary angiitis. J Ayub Med Coll Abbottabad.

[19] Vollmer TL, Guarnaccia J, Harrington W, Pacia SV, Petroff OA (1993). Idiopathic granulomatous angiitis of the central nervous system: diagnostic challenges. Arch Neurol.

[20] Pritchard C, Nadarajah K (2004). Tumour necrosis factor α inhibitor treatment for sarcoidosis refractory to conventional treatments: a report of five patients. Ann Rheum Dis.

